# Randomized, Single-Blind Comparison of Two Different Flow Rates of Sevoflurane Anesthesia on Acute Kidney Injury

**DOI:** 10.7759/cureus.80000

**Published:** 2025-03-03

**Authors:** Habib Md R Karim, Subrata K Singha, Chinmaya K Panda, Monica Khetarpal

**Affiliations:** 1 Anaesthesiology, Critical Care, and Pain Medicine, All India Institute of Medical Sciences, Guwahati, Guwahati, IND; 2 Anaesthesiology and Critical Care, All India Institute of Medical Sciences, Raipur, Raipur, IND

**Keywords:** drug-induced acute kidney injury, low-flow anesthesia, urinary-albumin-creatinine-ratio (uacr), urine microalbumin, volatile anesthetics

## Abstract

Background: Prolonged sevoflurane-based low-flow anaesthesia (LFA) is often not advised for clinical use due to acute kidney injury (AKI) risk. However, LFA has multitudes of advantages, and surveys on anesthesia practice indicate that the use of LFA, even with sevoflurane, is on the rise. Literature on human studies is growing, but the recommendations for LFA have not changed, indicating the need for further evaluation. We aimed to evaluate the incidence of AKI with sevoflurane-based LFA with two different FGFs.

Methods: The current prospective, randomized, single-blind, parallel-arm study was conducted using sevoflurane-based LFA with two FGFs: group A (1000mL/min) and group B (600mL/min) with a targeted age-adjusted minimum alveolar concentration (MACage) of 1-1.2 enrolling adult participants undergoing elective surgeries of at least 120-minute anesthesia duration. Anesthesia management was standardized, and AKI classification was performed based on Kidney Disease: Improving Global Outcomes (KDIGO) guidelines. Further, spot urinary microalbumin, sodium (Na), potassium (K), protein, and spot albumin creatinine ratio (sACR) were evaluated and compared. A two-tailed p-value <0.05 was considered statistically significant.

Results: Data from 65 (33 in the 1000mL and 32 in the 600mL group) were evaluated. No AKI was noted in either group. The anesthesia duration ranged from 120 to 780 minutes (median 200, interquartile range 260-180, with mean 230.9, and 95% confidence 202.5-259.3 minutes). Spot urine microalbumin was significantly higher at two to four hours postoperatively than at the preoperative level, but the rise was similar in both groups. By 24 hours, the level declined significantly and remained at a slightly higher level than the preoperative value, which further reduced to a somewhat lower level than the preoperative value by 48 hours. Only one patient in the 1000mL/min group had sACR >66.7 μg/mg.

Conclusion: Sevoflurane-based LFA with an FGF of 600mL/min is safe and comparable to the FGF of 1000mL/min for surgeries. Transient urinary microalbumin and sACR changes occur, which settle within 24 to 48 hours; no impact on urine output and AKI was noted.

## Introduction

The continually escalating healthcare costs worldwide are a big concern. Target-oriented healthcare delivery by prioritizing the treatments and interventions with minimized cost is one of the prime objectives that anesthesiologists must adopt [1, 2]. However, this cost-effective care should not compromise the patients' quality, evidence-based management, and safety [2, 3]. Low- and minimal-flow anesthesia has established its role in providing cost-effective, quality healthcare, leading to increasing acceptance by anesthesiologists [4, 5]. The low-flow technique is also associated with improved heat retention and humidity of inspired gases, potentially reducing the risk of respiratory tract reactivity. Further, volatile agents are greenhouse gases, and low-flow anesthesia (LFA) has environmental advantages [2,4]. Sevoflurane is a relatively newer inhalational agent widely used worldwide. However, it has been implicated in causing acute kidney injury (AKI) by producing compound A [6]. Compound-A production depends on the type of carbon dioxide absorbent, the amount of sevoflurane, and the fresh gas flow (FGF) used [7].

Nevertheless, although sevoflurane had a higher incidence of AKI than propofol and desflurane, compound A-induced AKI was neither associated with the loss of concentrating ability of kidneys nor associated with attributable increased mortality in human beings [6, 8]. Interestingly, the exposures to sevoflurane in these studies were for eight hours or so. Moreover, modern halogenated volatile anesthetics induce potent anti-inflammatory, anti-necrotic, and antiapoptotic effects that protect against ischemic AKI [9, 10]. The Food and Drug Administration (FDA) has recently lowered the limit of FGF used with sevoflurane, which, however, still recommends relatively higher FGF than in current practice [7, 11]. However, these recommendations are mainly based on the concentration of compound A produced with different flows and the evaluation of kidney function by estimating creatinine levels. Evidence suggests that urinary microalbumin level and microalbumin-to-creatinine ratio have been shown to detect AKI earlier than creatinine [12]. Therefore, a study evaluating AKI using readily available, low-cost biomarkers in sevoflurane-based LFA might provide better insights. A comparison of two different flows is also likely to help us make evidence-based decisions on sevoflurane-based low-flow and minimal-flow anesthesia. As breathing depends on the FGF, the toxic effect of sevoflurane might differ for different FGFs. Thus, the present study primarily aimed to quantify the incidence of AKI with sevoflurane-based LFA and compare it among two different flow rates, i.e., 600mL versus 1000mL/minute. We also assessed the 48-hour postoperative and 30-day self-reported morbidity and mortality as secondary objectives.

## Materials and methods

Settings and design

The prospective, randomized, single-blind, parallel-arm clinical study was conducted at All India Institute of Medical Sciences, an academic and research institute in Raipur, India, after due approvals from the institute ethics committee (letter no. 518/1EC-AIIMSRPR/2018 dated Nov 3, 2018, and extension letter number 2293/1EC-AIIMSRPR/2022 dated June 7, 2022). The institute's intramural project grant supported the study. Informed written consent was obtained from the participants for recruiting, and the study was prospectively registered with the Clinical Trial Registry of India (CTRI/2018/12/016500). Although the study planned to recruit participants from December 2018, the enrollment for the data collection commenced between July 2022 and May 2023.

Participants

Patients aged between 18 and 60 years, belonging to the American Society of Anesthesiologists (ASA) physical status class I and II of either male or female gender, having an estimated glomerular filtration rate (eGFR) > 60mL/minute/1.73 m² body surface area, undergoing elective non-cardiac surgeries were included. Patient refusal, patients of special groups (jail inmates, human immunodeficient patients, tribes protected under law), pregnant women, receiving contrast during surgery, continued intraoperative mean blood pressure < 60 mmHg for more than 10 minutes, and hypotensive anesthesia were excluded. The cases were also excluded if the anesthesia duration was less than 120 minutes.

Sample size

The study was planned as a pilot project for the initial part, with a total sample of 30 in two groups and a plan for an interim evaluation to calculate the final sample size if found suitable to continue safely. Further recruiting participants was planned to achieve an 80% power with an absolute precision of 5%. However, the interim analysis showed no AKI and much difference in the microalbumin level, necessitating a very high sample size (7677 per group) as calculated using the online epidemiological tool OpenEpi (The OpenEpi Project, Atlanta, GA; http://www.openepi.com) using the standard deviation (SD) difference at 24 hours, which was 0.8 (9.5 versus 8.7) between the groups [13]. Considering funding constraints, we planned to continue the study until the funds were available and recruited 68 participants.

Sampling technique

The research assistant screened all prospective participants for eligibility in the pre-anesthetic clinic. Eligible patients were counseled for participating in the study, and written and informed consent was obtained.

Randomization and blinding

Consenting participants were assigned into groups A and B. Patients were allocated randomly using a software-generated (online) block random number table. Randomization was done the day before a scheduled surgery. The group allocation was based on his/her block number and sequence, and the group mentioned against that code, which was on another sheet (paper) in table format and was concealed from the patient. The research assistant (a nursing officer recruited under the project) randomized and allocated the group, collected baseline data and sent samples for laboratory testing. Only the patient was blinded to the intervention. She also filled out the random code in the case record form and handed it to the anesthesia team. The anesthesiologist involved in case management was informed about the group over the phone just before the induction of general anesthesia (GA). The random codes were kept with her till the end of the recruitment to maintain concealment.

Intervention

The induction of GA was standardized and used titrated propofol dose, fentanyl 2μg/kg bolus, and vecuronium 0.1mg/kg as a muscle relaxant to facilitate tracheal intubation. All patients received sevoflurane, nitrous oxide, and oxygen-based anesthesia where the nitrous oxide expired fraction was maintained at 55 ± 5% (during maintenance). From the induction to achieving an equilibrium coefficient of 0.8, nitrous oxide was 60% on the dial side. The target age-adjusted minimum alveolar concentration (MACage) was kept between 1 and 1.2. Group A received an FGF of 1000 mL/min, and Group B received an FGF of 600 mL/minute during maintenance. Both groups underwent GA using the same model of anesthesia machine and the same type of carbon dioxide absorbent containing barium hydroxide as one component. Vecuronium was used to maintain muscle relaxation, predominantly guided by train-of-four monitoring. Any hypotension (Mean arterial pressure (MAP) < 60 mmHg) during the study period was treated using indirect or direct vasopressors, fluid, etc., as needed in both groups per institute practice and protocol. The entire patient's neuromuscular blockade was reversed using neostigmine, which was co-administered with glycopyrrolate.

All participants were kept fasted for a minimum of eight hours for solid fatty foods and were encouraged to drink water until two hours before surgery. In case fasting exceeded eight hours, maintenance intravenous fluids (Ringer's lactate) were usually started at approximately 80-100 mL/hour.

Protocol deviation/modification

The first version of the protocol included neutrophil gelatinase-associated lipocalin (NGAL) monitoring as outcome data. However, due to finance-related issues, the research cell and institute ethics committee later permitted the omission of NGAL. It was approved using the spot albumin creatinine ratio (sACR) and microscopy (letter no. 1500/IEC-AIIMSRPR/2021, dated February 16, 2021).

Outcome variables

Demographic and clinical parameters, ASA physical status, creatinine, blood urea, duration of anesthesia, mean blood pressure (MBP), creatinine, blood urea, and intraoperative urine output (UO) were noted. Further, urine spot microalbumin, spot protein, spot sodium, and spot potassium levels were noted preoperatively, between two to four hours postoperatively, 24 hours postoperatively, and 48 hours postoperatively. Furthermore, urine testing and microscopy were done to detect distorted red blood cells, casts, and myoglobinuria simultaneously.

Follow-up

We planned a 30-day follow-up to evaluate the urine-related problems and biochemical changes and requested to attend the hospital. If the participants did not attend the appointment, a telephone interview was planned to ask about any problems with urine. 

Data management

The research assistant entered data in Microsoft Excel (Microsoft Corporation, Redmond, WA, United States) from the case record form and searched the electronic investigations database through the institute app to prepare the master chart. The primary investigator verified the hypotension categorization further per the hypotension definition and revised it as required. Intraoperative hypotension was defined as an MBP of <60 mmHg or even if the MBP was >60 mmHg, but the fall from the baseline was >30% [14]. Intraoperative UO data was unavailable for three patients (two from group A and one from group B). The average UO per minute of anesthesia was calculated from the available data, and the missing three data were filled with the calculated value as (average UO per minute x anesthesia duration). Ten patients' microalbumin levels were reported as ‘<5’. We filtered all patients whose reported microalbumin levels were less than five, but exact values were available, and averaged them to convert the ‘<5’ value to the average. The categorical data were calculated using absolute numbers and percentage scales as required for further analysis. Intraoperative UO and crystalloid infusion volume were also converted to mL/kg, and urinary spot microalbumin to sACR was calculated and compared. The Kidney Disease: Improving Global Outcomes (KDIGO) classification for AKI was used to diagnose AKI among the participants [15].

Statistical analysis

Statistical data analysis was done using InStat software (GraphPad Software, Inc., La Jolla, CA, United States) with appropriate statistical tests based on the data distribution as tested by the k-test. Categorical data are presented as median and interquartile range; continuous data are presented as mean and standard deviation. P-values were calculated as two-tailed, and values < 0.05 were considered significant.

## Results

**Figure 1:**
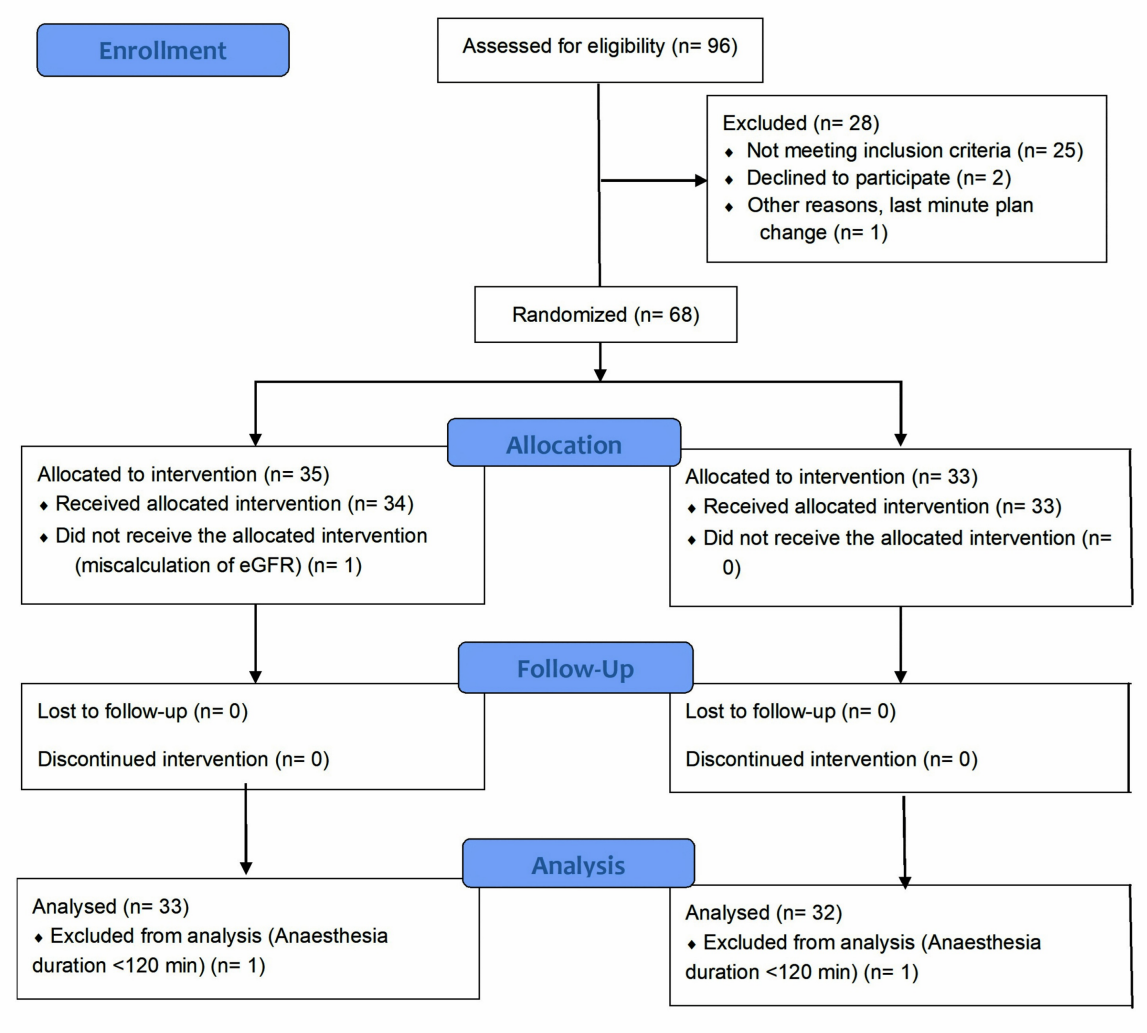
Consolidated Standards of Reporting Trials (CONSORT) 2010 flow diagram FGF: fresh gas flow; eGFR: estimated glomerular filtration rate

Ninety-six participants were screened for eligibility, and following the exclusions and follow-up loss, data from 65 participants (33 in the 1000mL/min group, 32 in the 600mL/min group) were analyzed. Figure 1 shows the Consolidated Standards of Reporting Trials (CONSORT) 2010 flow diagram for the present study [16].

Both groups had a female majority: 18 (54.55%) and 22 (68.75%), respectively. The groups' demographic distribution, ASA physical status, surgical invasiveness grades, eGFR, and preoperative basic hemodynamic parameters were statistically indifferent (Table 1).

**Table 1:** Clinico-demographic and preoperative parameters tested using Fisher's exact test and *unpaired t-test. Group A denotes 1000mL/min and Group B denotes 600mL/min. NICE: National Institute for Health and Care Excellence; ASA: American Society of Anesthesiologists; BSA: body surface area; eGFR: estimated glomerular filtration rate; METs: metabolic equivalents of tasks

Parameters	Group A (N=33)	Group B (N=32)	p-value
Male/Female (number, %)	15 (45.45) / 18 (54.55)	10 (31.25) / 22 (68.75)	0.310
Age (years)	37.91 ± 12.67	41.0 ± 10.99	0.298^*^
Height (cm)	160.64 ± 8.55	157.22 ± 9.77	0.138^*^
Weight (kilogram)	57.45 ± 9.88	58.06 ± 8.88	0.795^*^
Body mass index (kg/m^2^)	22.41 ± 2.79	23.33 ± 3.08	0.212^*^
Comorbid patients (number, %)	6 (18.18)	10 (31.25)	0.260
NICE surgical grade 2/3/4 (number, %)	7 (21.21) / 20 (60.61) / 6 (18.18)	14 (43.75) / 11 (34.38) / 7 (21.87)	0.082
ASA-PS class I/II (number, %)	14 (42.42) / 19 (57.58)	9 (28.13) / 23 (71.87)	0.302
METs category: good /intermediate (number, %)	32 (96.97) / 1 (3.03)	27 (84.38) / 5 (15.62)	0.105
Preoperative eGFR (mL/1.72 m^2^ BSA)	95.15 ± 20.08	90.94 ± 28.25	0.534^*^
Baseline mean blood pressure (mmHg)	87.61 ± 9.96	84.25 ± 10.95	0.201^*^
Baseline heart rate (per minute)	79.06 ± 12.12	80.19 ± 12.24	0.711^*^

Ten (30.3%) cases in group A (1000mL/min FGF) and eight (25.0%) in group B (600mL/min FGF) had episodes of hypotension, which was treated using phenylephrine 25 mcg or mephentermine 3mg bolus and 100-200mL fluid rapid infusion. None of the patients had hypotension lasting more than 10 minutes. The anesthesia and surgery durations, intraoperative fluid administration, and urine output were also comparable among the groups (Table 2).

**Table 2:** Intraoperative anesthesia-management-related data compared between the groups using an unpaired t-test and *Fisher's exact test. Group A denotes 1000mL/min and Group B denotes 600mL/min.

Parameters	Group A (N=33)	Group B (N=32)	p-value
Lowest mean blood pressure (mmHg)	69.97 ± 9.96	69.97 ± 12.62	0.868
Hypotension (number and %)	10 (30.3)	8 (25.0)	0.782^*^
Anesthesia duration (min)	229.7 ± 189.5	233.1 ± 140.2	0.907
Surgery duration (min)	201.9 ± 84.1	206.1 ± 129.2	0.878
Total urine output (mL)	391.67 ± 126.99	395.31 ± 186.39	0.926
Urine output mL/kg/h	1.93 ± 0.63	2.06 ± 1.2	0.564
Total crystalloid mL	954.5 ± 252	1157.8 ± 543.9	0.058
Crystalloid mL/kg/h	4.84 ± 1.82	5.78 ± 2.59	0.094

**Figure 2:**
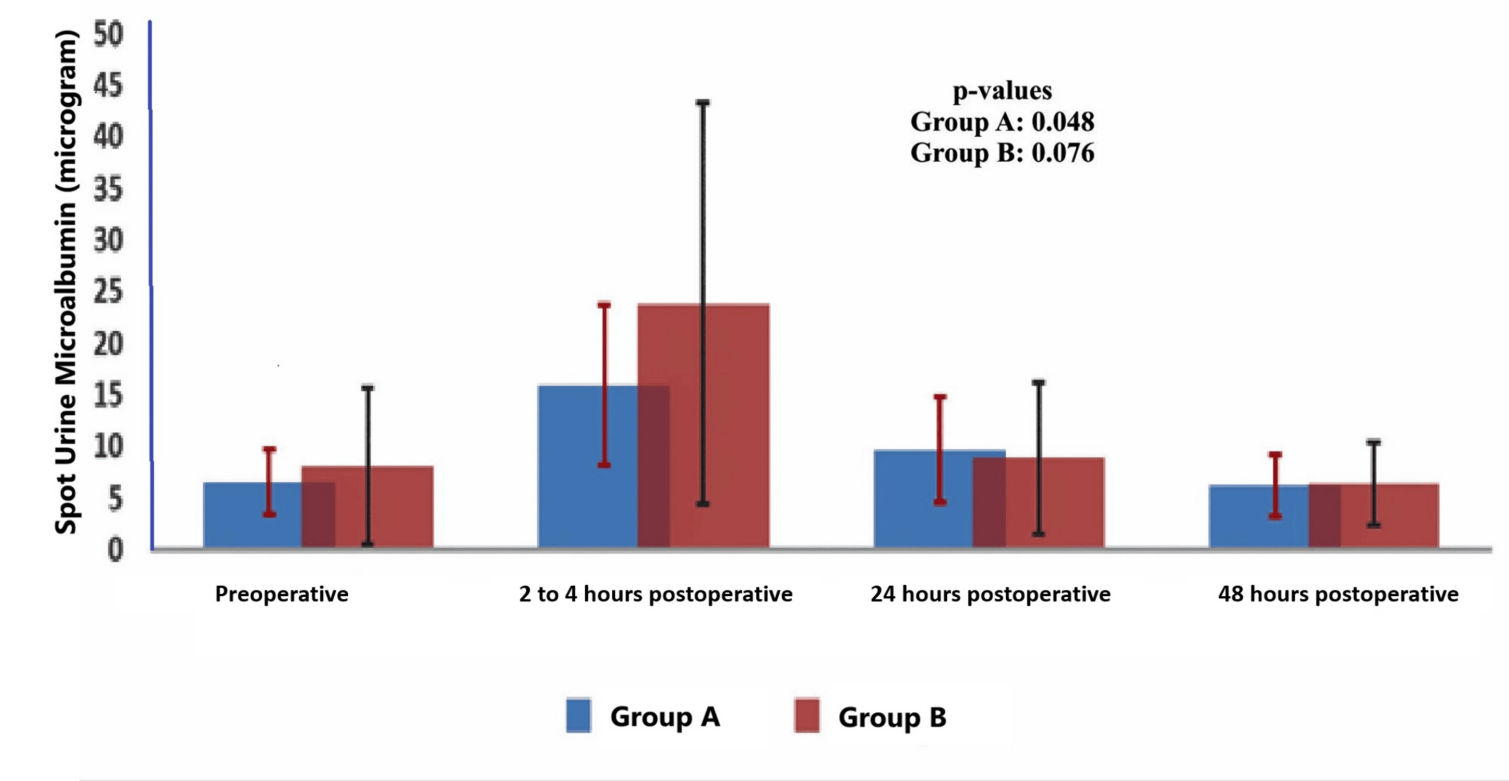
Mean and 95% confidence of urine spot microalbumin at different time points. The intergroup p-values were <0.05 (unpaired t-test). The p-value mentioned in the figure is for intra-group variation (analysis of variation test). Group A denotes 1000mL/min and Group B denotes 600mL/min.

Due to technical difficulties, postoperative two- to four-hour samples and testing were missed in 31 (47.69%) cases. Therefore, the data for that time point are not presented in detail, but when compared between the groups with limited data, the differences were insignificant at each time point. However, analysis of the data from 34 participants as a single cohort and comparison showed that the urine microalbumin at two to four hours was significantly higher than the preoperative level (7.08 ± 15.61 versus 18.79 ± 26.87; p-value 0.006) and even higher than 24 hours (18.79 ± 26.87 versus 9.27 ± 17.26; p-value 0.032). The analysis also showed that the variation (rise from the preoperative level and fall to the 24-hour level) was statistically significant; p-value 0.012. When intragroup variations were compared, the changes in the different time points were statistically significant for Group A and Group B. The mean and 95% confidence of urine microalbumin are presented in Figure 2.

A comparison of the microalbumin, spot sodium, spot potassium, protein levels, serum creatinine, and blood urea obtained preoperatively, 24 hours, and 48 hours postoperatively showed that the intergroup and intragroup changes were not statistically significant. Although there was a slight increase in the sACR level at 24 hours postoperatively compared to the preoperative level, the differences were statistically insignificant for both groups, and the level reached the preoperative level by 48 hours. Both inter-group and intra-group changes were similar (Table 3).

**Table 3:** Urine biochemical tests and derived data were compared between the group using an unpaired t-test and an intra-group comparison (@) for preoperative versus 24-hour; it was tested using a paired t-test and for preoperative (#) versus 24-hour versus 48-hour, it was tested using analysis of variation (ANOVA). sACR: spot urine albumin and serum creatinine ratio

Parameters	Groups	Preoperative	P-value	24 hours postoperatively	P-value	P-value^@^	48 hours postoperatively	P-value	P-value^#^
Urine spot microalbumin (μg/dl)	A	6.51 ± 8.8	0.687	9.7 ± 14.6	0.851	0.286	6.22 ± 6.3	0.946	0.899
	B	8.11 ± 21.05		8.8 ± 20.36		0.883	6.38 ± 9.05		0.718
Urine spot sodium (meq/dl)	A	78.67 ± 38.75	0.964	78.03 ± 40.51	0.853	0.948	75.21 ± 60.61	0.939	0.793
	B	79.16 ± 48.28		79.94 ± 42.35		0.945	74.15 ± 37.76		0.663
Urine spot potassium (meq/dl)	A	27.27 ± 18.55	0.741	26.36 ± 17.36	0.469	0.837	16.79 ± 13.77	0.951	0.023
	B	25.81 ± 16.94		23.44 ± 14.91		0.553	17.0 ± 10.59		0.022
Urine spot protein (mg/dl)	A	5.82 ± 4.5	0.405	7.55 ± 6.14	0.601	0.195	6.52 ± 4.59	0.523	0.589
	B	7.0 ± 6.7		6.82 ± 4.98		0.904	8.30 ± 11.63		0.603
Serum creatinine (mg/dl)	A	0.81 ± 0.17	0.638	0.88 ± 0.18	0.488	0.112	0.88 ± 0.16	0.118	0.086
	B	0.83 ± 0.21		0.85 ± 0.09		0.593	0.82 ± 0.11		0.894
Blood urea (mg/dl)	A	19.52 ± 4.87	0.227	23.12 ± 9.81	0.916	0.063	20.25 ± 7.71	0.604	0.686
	B	21.34 ± 7.06		23.34 ± 6.93		0.257	21.75 ± 8.47		0.861
sACR (μg/mg)	A	8.06 ± 10.76	0.923	11.22 ± 17.39	0.768	0.378	6.46 ± 6.62	0.801	0.408
	B	8.39 ± 16.53		9.83 ± 20.35		0.119	7.22 ± 11.47		0.858

Only one patient's urine microscopy at the two- to four-hour postoperative period in the 600mL/min group showed distorted red blood cell (RBC) count, but the 24-hour and 48-hour postoperative urine did not show anything. None of the patients in both groups showed myoglobinuria at any tested time points.

## Discussion

The present study showed that sevoflurane-based LFA, even with 600mL/min of FGF at a target MACage of up to 1.2 with nearly 60% nitrous oxide for an anesthesia duration ranging from 120 to 780 minutes with a mean duration of 230.9 minutes and a 95% confidence limit of 202.5 to 259.3 minutes, was safe in context to cause renal impairment. If converted to minimum alveolar concentration*time in hours (MAChour) for only sevoflurane, 260 minutes of anesthesia will be equivalent to approximately 2.47 (1.2 MAC x (60% fraction of inspired nitrous oxide / one MAC of nitrous oxide, i.e., 104%) x 4.33 hours). None of our patients had low urine output during the intraoperative and postoperative periods. The creatinine and blood urea levels were also normal, and the rise was not significant, classifying them as AKI per the definition provided by KDIGO [15]. The urinary sACR is very effective in predicting AKI, even having a better predictive value (area under the curve 0.725; 95% confidence interval 0.676-0.774) than NGAL and plasma cystatin-C with a cut-off value of ≥ 66.7 μg/mg as having the best diagnostic accuracy [17]. Only one patient in the 1000mL/min FGF showed a urinary sACR level above this cut-off. However, the patient did not fall into the AKI classification as per KDIGO.

Our finding echoes the findings of a few other studies, where the risk of AKI was not noted with sevoflurane-based LFA, even with the use of biomarkers [6, 18, 19]. However, a survey conducted by Australian and New Zealand anesthesiologists shows that the FGF used for sevoflurane-based anesthesia is still > 1000mL/min [20]. The secondary analysis of the data of an Indian survey on the practice of low and minimum-flow anesthesia indicates that only 5.1% used FGF < 600 mL/min and 19.1% used 600-1000 mL/min; use of <1000mL/min FGF was more with desflurane [11]. These practices might reflect the impact of the recommendations made by the FDA, which recommends using sevoflurane with an FGF of 1 - <2 L/min and up to two minimum alveolar concentration (MAC) hours. The FDA recommends using FGF >2 L/min beyond two MAC hours [7]. Even the ASA recognizes the need for LFA for sevoflurane and indicates no or negligible risk of AKI with sevoflurane-based LFA; the society, however, still does not make any recommendation on the lower limit of FGF to be used [21].

Although we did not include cardiac surgical patients, who have a high incidence of postoperative AKI [22], a study using minimal flow (500 mL/min FGF) anesthesia with sevoflurane found no difference in the postoperative cardiac-surgery-associated AKI in on-pump surgeries [23]. Our 600mL/min FGF arm also did not show any case of AKI; this suggests that a minimal flow of 500-600 mL/min is feasible with sevoflurane-based anesthesia without a significant risk of AKI in human beings whose preoperative kidney functions were normal (eGFR >60 mL/min). The findings of the present study and others, as discussed above, indicate that the FDA and ASA should revise their recommendations and recommend using Sevoflurane-based LFA with FGF <1000mL/min.

Our study has a few limitations that also need discussion. Firstly, the sample size is inadequate to have a desired acceptable power. Further, a few patients were discharged from the hospital on the second day before the 48-hour sample. Follow-up on the 30^th^ day was possible only through a telephone conversation, where none of the patients complained about urine issues and low output. Biochemical testing follow-up was not available as patients did not attend the hospital. In a few cases, UO monitoring data was available on-shift-wise, as and when voided, and in approximate amounts for postoperative days in most of the cases. Although none of the cases had poor urine output, the precise calculation was not feasible, so the comparison was not made. Further, the present single-center study included low-risk patients; most were non-comorbid. Even the hypertensive patients were well-controlled. Therefore, the results can be generalized, especially for the mentioned groups. Although we follow standard practice for preoperative fasting and starting intravenous fluid in fasted patients, we have not compared them among the group. Further, the present study also used nitrous oxide and sevoflurane with only oxygen, which might have a different impact. Previous studies have shown that compound A can impact different parts of the nephron, and UO is usually maintained [6]. The KDIGO uses UO as one of the criteria, which also might impact the interpretation.

## Conclusions

The study results suggest that sevoflurane-based LFA with an FGF of 600mL/min is safe and comparable to the FGF of 1000mL/min for surgeries where anesthesia duration exceeds MAChour 2. Sevoflurane-based LFA did not cause AKI, and there were no significant changes in the urinary biochemical testings. None of the patients had low urine output till the 48-hour postoperative period. Although the urinary microalbumin was raised at the two- to four-hour postoperative period and 24 hours, they were back to the preoperative level within 48 hours. The same was also noted for urine sACR. Only one patient in the 1000mL/min FGF showed to be at risk for AKI as per urine sACR value but the patient did not have AKI as per KDIGO.
